# Target antigen screening and development of a multi-component subunit vaccine against *Mycoplasma synoviae* in chickens

**DOI:** 10.3389/fcimb.2024.1458865

**Published:** 2024-10-23

**Authors:** Xiaomei Sun, Mingyong Deng, Chuxing Cheng, Ya Zhao, Zuqing Liu, Yu Yang, Qiaoxia Xu, Rong Yao, Min Hu, Meilin Jin, Chao Kang

**Affiliations:** ^1^ National Key Laboratory of Agricultural Microbiology, Huazhong Agricultural University, Wuhan, China; ^2^ College of Animal Medicine, Huazhong Agricultural University, Wuhan, China; ^3^ The Cooperative Innovation Center for Sustainable Pig Production, Wuhan, China; ^4^ Research Institute of Wuhan Keqian biology Co., Ltd, Wuhan, China; ^5^ Hubei Province Animal Disease Comprehensive Prevention and Control Industry Technology Innovation Alliance, Wuhan, China; ^6^ Hubei Jiangxia Laboratory, Wuhan, China

**Keywords:** *Mycoplasma synoviae*, subunit vaccine, MSPB, Ppht, Cfba, EF-G

## Abstract

**Introduction:**

*Mycoplasma synoviae* (MS) is a globally important avian pathogen causing infectious synovitis and respiratory diseases in poultry, leading to significant economic losses. Despite advances in vaccine development, a commercially viable subunit vaccine against MS remains elusive.

**Methods:**

We sequenced whole genomes of six clinical MS strains isolated from different Chinese provinces. Common genes were analyzed using Biopython software, identifying those with high copy numbers in virulent strains and shared among all strains. Vaxign2 and IEDB Antibody Epitope Prediction were used to analyze protein properties. We assessed immune protective effects of candidate proteins and developed a multivalent subunit vaccine.

**Results:**

Ten candidate vaccine proteins were initially selected. A multivalent subunit vaccine composed of MSPB, Ppht, Cfba, and EF-G displayed the best protective effect. The optimal immunization dosage was 20μg, with each protein accounting for 25%. The immune production period was determined to be 28 days post-first immunization, lasting 180 days. The immune protection rate against highly virulent strains reached 90%∼100%.

**Discussion:**

This study provides a new approach for screening vaccine antigens and develops an effective candidate vaccine for MS prevention. The multivalent subunit vaccine shows promising results in protecting against MS infections, potentially offering a solution to reduce economic losses in the poultry industry.

## 1 Introduction

*Mycoplasma synoviae* (MS) is a pathogen that poses a substantial threat to the poultry industry worldwide, has been isolated in Europe, Asia and Africa and United States (Feberwee et al., 2022). MS could infect chickens, turkeys, geese, ducks, ostriches, partridges, quail, pheasants, guinea fowl and wild birds (Lierz et al., 2008; Michiels et al., 2016; Sawicka et al., 2020).MS infections can induce clinical symptoms such as respiratory tract infections, synovitis, and the swelling of joints and tendon sheaths in poultry (Feberwee et al., 2009; Sun et al., 2017; Xue et al., 2017; Bergeron et al., 2021; Sui et al., 2022; Wei et al., 2023), reduce egg production of up to 23%, and cause eggshell apex abnormalities (EAAs) (Feberwee et al., 2009).Owing to its capacity for both horizontal and vertical transmission, eradicating MS is a formidable challenge (Ter Veen et al., 2020). Furthermore, persistent MS infections significantly compromise the resistance of a flock, rendering it highly susceptible to secondary infections by other pathogenic microorganisms, thereby increasing the risk of animal mortality (Xavier et al., 2011). Consequently, MS infections not only present significant difficulties in disease prevention and control but also inflict substantial economic losses on the poultry industry.

MS infection control currently relies primarily on comprehensive measures, including enhanced production management of poultry farms, source purification, medication, and vaccination for immunity. Purifying breeder flocks incurs high costs, and establishing and maintaining MS-free breeder flocks is challenging, making this method less applicable to smaller-scale breeder farms. Although antibiotics have preventive and therapeutic effects against MS (Dufour-Gesbert et al., 2006; Hong et al., 2015), the increasing incidence of antibiotic resistance adds complexity to disease control (Kreizinger et al., 2017; Beylefeld et al., 2018; Emam et al., 2020; de Jong et al., 2021; Zhang et al., 2022; Poudel et al., 2024). Research has suggested that traditional Chinese medicines or plant extracts also possess inhibitory effects against MS. Moreover, combining traditional Chinese and Western Medicine can reduce the development of MS resistance and alleviate clinical symptoms (Awad et al., 2023). However, this method does not eliminate MS infections in poultry, making it a non-long-term solution.

Therefore, vaccination remains the most effective means of preventing the occurrence of the disease and halting the transmission of MS. The research and development of efficient vaccines have become crucial in MS studies in recent years. Currently two live attenuated vaccines are commercially available, the temperature-sensitive MS-H vaccine and the NAD-independent MS1 vaccine (Markham et al., 1998; Noormohammadi et al., 2003; Jones et al., 2006b; a; Noormohammadi et al., 2007; Shahid et al., 2013; Feberwee et al., 2017; Liao et al., 2024). However, the live attenuated vaccines are expensive and require stringent storage conditions. Moreover, they are not suitable for poultry that are already infected with MS. Forced vaccination can exacerbate the clinical symptoms of MS-positive poultry, leading to greater economic losses. Consequently, the widespread application of live attenuated vaccines are greatly restricted to countries and regions with high MS positivity rates. Additionally, inactivated vaccines are commonly used for MS (Gong et al., 2020; Marouf et al., 2022). Although these vaccines can stimulate an organism to produce good immune responses and reduce the infection rate of MS, weak cross-protection between different prevalent MS strains in various regions and issues such as long culture times and high culture costs during vaccine production severely limit the application of this type of vaccine. In contrast to live attenuated or inactivated vaccines, subunit vaccines comprise only specific immunogenic components of the pathogen rather than the entire organism. This approach eliminates the risk of reversion to virulence, thereby offering enhanced safety and stability profiles compared to whole-cell formulations (Baxter, 2007). However, despite these advantages, no commercially available subunit vaccine against MS has yet been developed, still in the laboratory research stage (Zhang et al., 2023). Therefore, the urgent need for the development of safe, broad-spectrum, and efficient vaccines remains crucial for preventing and controlling MS.

In this study, we conducted whole-genome sequencing of six MS strains isolated from different regions in China, each exhibiting significant differences in pathogenicity. Using Biopython software, we performed a comparative analysis of 23 MS strains available in the NCBI database. We then identified proteins shared among all strains and those with high copy numbers in virulent strains as potential antigen targets. Finally, by comparing the immunoprotective effects of single-antigen, dual-antigen, and multi-antigen combinations, we successfully developed a safe, broad-spectrum, and efficient multi-component subunit vaccine against MS. Hence, this study not only provides new insights into the development of novel MS vaccines but also offers potentially effective means for MS prevention and control.

## 2 Materials and methods

### 2.1 Ethics statement

All animal experiments were performed at Wuhan Keqian Biological Co., Ltd. All experimental procedures conformed to the guidelines of the Hubei Province Animal Regulations, in accordance with international law. The approval number for the ethical review of experimental animal welfare is SOR-AR-022.

### 2.2 Sample sources

Six MS strains isolated and identified in the laboratory were collected from 2019 to 2020 from five provinces in China, namely, Hubei, Jiangxi, Henan, Anhui, and Hunan.

### 2.3 Pathogenicity assay of the six strains

The six isolated MS strains were subjected to artificial infection experiments. Briefly, each strain was inoculated at a dose of 0.5 mL with a live bacterial content of 1.0 × 10^7^ CCU/mL. Five specific-pathogen-free (SPF) chickens (35∼38 days old) were inoculated with each strain. After 14 days of artificial infection, If two or more of the following four conditions are present: limping, swelling in the palm of the foot, swelling in the tarsal joint, or swelling in the toe joint, it is considered an onset of the disease. The pathogenicity of the different strains in chickens were compared according to the incidence rate of each group.

### 2.4 Establishment of a bacterial challenge model

The highly virulent strain was selected as the challenge strain for subsequent experiments and a chicken challenge model of MS infection was established. After determining the live bacterial content, a suspension of the highly virulent strain was adjusted to a bacterial density of approximately 1.0 × 10^7^ CCU/mL in a modified Frey liquid medium. Next, 40 SPF chickens (35∼38 days old) were randomly selected, with 30 chickens divided into three infection groups (n=10 each group), and the remaining 10 were used as the blank control group. Each chicken in the infected group was injected with 0.1, 0.2, or 0.3 mL of bacterial suspension through foot pad injection, while the control group was injected with 0.3 mL of modified Frey liquid culture medium. After 14 days of artificial infection, The pathogenicity of the different groups were compared according to the incidence rate of each group. The criteria for determining the onset of the disease are described in section 2.3.

Fourteen days post-infection, the incidence of illness in the experimental chickens was recorded. Joint fluid was collected from the tarsal, hock, and toe joints to isolate live bacteria. The joint fluid was then inoculated into a modified Frey liquid medium and incubated at 37°C with agitation for 24h. After incubation, the culture medium was filtered through 0.22μm filter membrane. The filtrate was then inoculated into a modified Frey liquid medium and incubated without shaking at 37°C for 7 days. A color change from crimson to orange in the culture medium indicated the presence of MS bacterial cells. The optimal number of live bacteria for the virulent MS strain in 35∼38-day-old SPF chickens was then determined.

### 2.5 Genomic DNA isolation and sequencing

The MS strains were inoculated into a mycoplasma culture medium containing 10% swine serum and 0.01% (w/v) nicotinamide adenine dinucleotide and grown at 37°C until the late logarithmic phase (pH = approximately 6.8) at a final volume of 50 mL. The bacterium was then collected via centrifugation at 10000 rpm for 30 min. Finally, genomic DNA was prepared via CTAB extraction and ethanol precipitation.

Whole-genome sequencing of the six MS strains using the PacBio Sequel II platform was conducted by Wuhan Frasergen Co., Ltd. Raw sequencing data were assembled using Microbial Assembly (smrtlink8), while HGAP4 software (smrtlink8) and Canu (v1.6) were used to assemble pure third-generation data.

### 2.6 Analysis of whole genome sequences and screening of candidate vaccine antigens

The shared genes of the six MS strains were analyzed using Biopython software to identify proteins present in high copy numbers in the highly virulent strains. These proteins were considered potential antigen targets. The sequences of these potential antigens were compared to existing MS whole-genome sequences in the NCBI database to verify their broad-spectrum identity. Subsequently, Vaxign2 and IEDB antibody epitope predictions were used to analyze the properties of these proteins and select potential vaccine candidates.

### 2.7 Construction of recombinant plasmids and protein expression

Candidate proteins were synthesized based on their full-length gene sequences, and the codons of each gene were optimized before cloning into expression vectors. The successfully constructed recombinant plasmids were transformed into *Escherichia coli* BL21(DE3) cells, and protein expression was induced using IPTG (final concentration, 0.4 mmol/L) at 18°C for 12 h. After induction, the bacterial cells were collected via centrifugation, resuspended in a buffer, and disrupted using pressure. The cell lysate was centrifuged at 12000 rpm, 4°C for 30 min. If the target protein was soluble, the supernatant was collected and purified using nickel affinity chromatography. The inclusion body extraction method was used if the target protein formed inclusion bodies. The proteins in the precipitate were denatured and refolded to obtain the target protein.

### 2.8 Reaction of candidate antigens with MS-positive serum and MS- negative serum

Serum from SPF chickens infected with MS (MS-positive serum) and serum from SPF chickens (MS- negative serum)were collected as primary antibody, and HRP-conjugated goat anti-chicken IgG was used as the secondary antibody. Western blot analysis was performed on the expressed candidate proteins to screen for positive proteins as candidate vaccine antigens.

### 2.9 Validation of the immunoprotective efficacy of individual proteins

The candidate proteins were adjusted to a concentration of 400 μg/mL and emulsified with Freund’s complete adjuvant and Freund’s incomplete adjuvant in equal volumes to prepare the first and second immunization vaccines, respectively. The protein concentration in the vaccines was 200μg/mL.

SPF chickens aged 7∼10 days were randomly divided into twenty-one groups, with 10 chickens in each group. For the first immunization, 0.1 mL or 0.2 mL of Freund’s complete adjuvant was subcutaneously injected into the neck of each chicken. Fourteen days after the first immunization, Freund’s incomplete adjuvant vaccine was administered using the same immunization method and dosage. Meanwhile, a control group treated with the adjuvants alone and a negative control group treated with modified Frey liquid culture medium were also established. Fourteen days after the second immunization, the immunized, adjuvant control, and negative control groups were challenged. The protective efficacy of each protein immunization group was evaluated at 14 days post-challenge. If none of the following four conditions exist: limping, palm swelling, tarsal joint swelling, or toe joint swelling, it is considered to have successfully obtained immune protection.

### 2.10 Validation of the immunoprotective efficacy of dual and multiple protein combination vaccines

The highly protective proteins were selected, and different combinations of two or more antigens were tested. Each protein in the vaccine had a final concentration of 100 μg/mL, and the immunization dosage was 0.2 mL per chicken. The methods of immunization, infection, and assessment of protective effects are as described in section 2.9. The combination of antigen proteins with the best immunization efficacy was identified.

### 2.11 Enzyme-linked immunosorbent assay

Antibody was tested by indirect ELISA with the Candidate proteins respectively and peroxidase conjugated goat anti-chicken IgG (Southern Biotechnology Associates). Briefly, ELISA plates were coated over-night at 4°C with Candidate proteins respectively protein (1μg/ml, 100μL per well). After blocked with PBS containing 5% skim milk for 2h at 37°C, the plates were added with sera at a dilution of 1: 800. After incubation for 30 min at 37°C, the plates were washed five times with washing buffer (PBS containing 0.05% Tween-20). A 1:20,000 diluted anti-chiccken IgG was added and incubated for an additional 30 min. After another 5 washes, TMB Substrate (Sigma-Aldrich, USA) was added and incubated for 15 min. Then the reaction was stopped, and optical density (OD) was measured at 630 nm. Those sera were considered positive if the OD values were higher than the mean OD of the 60 sera collected from SPF chickens.

### 2.12 Study on antibody dynamics and duration of immunity

Eighty SPF chickens aged 7∼10 days were randomly divided into eight groups, with 10 chickens in each group. Groups 1∼4 were immunized with the multi-component subunit vaccine via subcutaneous injection in the neck and back at a dosage of 0.2 mL. Fourteen days after the first immunization, the second immunization was administered through the same route and at the same dosage. Groups 5∼8 were served as the non-immunized control groups.

The antibody levels in the immunized groups were monitored. Groups 1 and 2 underwent wing vein blood collection at 7, 14, 21, and 28 days post-primary immunization. Groups 3 underwent wing vein blood collection at 7, 14, 21, 28, 35, 60, 90, 120, 150, and 180 days post-primary immunization, and Groups 4 underwent wing vein blood collection at 7, 14, 21, 28, 35, 60, 90, 120, 150, 180, and 210 days post-primary immunization. The collected serum samples were subjected to ELISA to measure the antibody levels against each protein.

Groups 1∼4 and 5∼8 were challenged with fresh MS bacterial suspensions via footpad injection at 7 days (groups 1 and 5), 14 days (groups 2 and 6), 166 days (groups 3 and 7), and 196 days (groups 4 and 8) after the second immunization. The dosage of the bacterial suspension was 0.3 mL, with a live bacterial content of approximately 3.0 × 10^6^ CCU.

### 2.13 Analysis of tissue pathological changes

The lung and trachea were collected from the chickens in the challenge group after the second immunization. Hematoxylin and eosin (HE) staining was performed on these tissues to compare and analyze pathological changes in the organs of immunized and control groups after bacterial challenge.

## 3 Results

### 3.1 Pathogenicity study of the six MS strains

The chicken challenge experiment indicated that the HB03 strain exhibited the highest virulence. Fourteen days after inoculation, all five chickens showed signs of illness, with swelling in the footpad and tarsal joints, accompanied by the presence of caseous material upon dissection. Pathogen isolation results were positive. The other five MS strains were capable of causing varying degrees of illness, but their pathogenicity was lower than that of HB03 (**Table 1**).

**Table 1:** Artificial infection test of 6 *M. synoviae* strains.

Team	Source	Incidence rate	Pathogen isolation rate
HB01	Jiangxi	60%	60%
HB02	Anhui	60%	60%
HB03	Hubei	100%	100%
HB04	Hubei	40%	40%
WH01	Henan	60%	60%
WH02	Hunan	20%	20%
Negative control	Modified Frey liquid culture medium	0.00%	0%

### 3.2 Determination of challenge dose and establishment of challenge model

The highly virulent HB03 strain was used to establish a chicken challenge model. When challenged with a dose of 0.1 mL per chicken, the rates of footpad swelling, tarsal joint swelling, and toe joint swelling at 14 days post-infection were 60%, 10%, and 10%, respectively. The overall morbidity and pathogen isolation rates at this dose were 70% and 60%, respectively. When challenged with a dose of 0.2mL per chicken, the rates of footpad, tarsal joint, and toe joint swelling at 14 days post-infection were 70%, 20%, and 20%, respectively. The overall morbidity and pathogen isolation rates at this dose were 80% and 70%, respectively. Finally, when challenged with a dose of 0.3 mL per chicken, the rates of footpad swelling, tarsal joint swelling, and toe joint swelling at 14 days post-infection were 90%, 30%, and 20%, respectively. The overall morbidity and pathogen isolation rates at this dose were 100% and 90%, respectively (**Table 2**). Based on these results, a dose of 0.3 mL was selected as the optimal challenge dose for SPF chickens aged 35∼38 days.

**Table 2:** Different challenge dose and morbidity of HB03.

Challenged Dose (mL)	days	Pathogenic site and morbidity		overall morbidity (%)	pathogen isolation rates (%)
		Pathogenic site	morbidity (%)		
0.1	14	Swollen foot pad	60	70	60
		Swollen tarsal joint	10		
		Swollen toe joint	10		
0.2	14	Swollen foot pad	70	80	70
		Swollen tarsal joint	20		
		Swollen toe joint	20		
0.3	14	Swollen foot pad	90	100	90
		Swollen tarsal joint	30		
		Swollen toe joint	20		
Negative control	14	Swollen foot pad	–	0	0
		Swollen tarsal joint	–		
		Swollen toe joint	–		

### 3.3 General comparisons of the six MS genomes

The genome sizes of the six strains are about 1.0 MB, with gene numbers varying from 1313 to 1401, and GC content ranging from 28.34% to 28.65% (**Table 3**). HB03 had the largest genome size and the highest number of genes among the six isolated strains.

**Table 3:** General characteristic of the genomes of 6 *M. synoviae* strains.

Sample Name	Length	All Reads	Aligned Reads	Unaligned Reads	Aligned Base	Average Depth	Coverage
HB01	780,886	82,010	80,869	1,141	773,547,187	990.60X	100.00% (780,886)
HB02	791,254	99,095	97,896	1,199	927,732,590	1172.48X	100.00% (791,254)
HB03	828,800	143,186	135,538	7,648	1,078,006,809	1300.68X	97.71% (809,860)
HB04	815,866	75,447	55,700	19,747	525,527,585	644.13X	100.00% (815,866)
WH01	774,030	232,504	229,792	2,712	2,086,097,671	2695.11X	100.00% (774,030)
WH02	777,124	306,751	305,147	1,604	2,795,218,397	3596.88X	100.00% (777,124)

### 3.4 Selection of immunogenic protein-encoding genes

**Figure 1:**
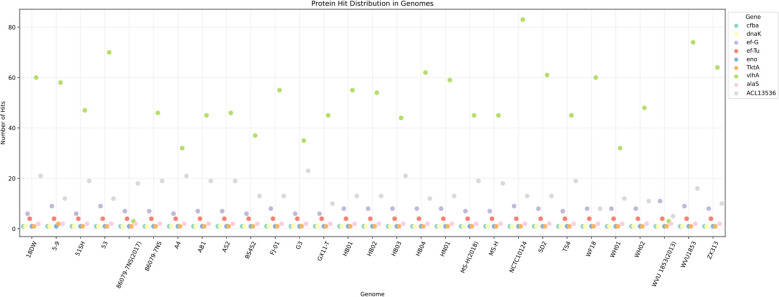
Distribution of 9 candidate genes in Mycoplasma synovialis strains in the NCBI database. Sequence alignment revealed that these nine genes were present in all 23 strains in the NCBI database.

The copy numbers of all genes in the genomes of the six strains were analyzed by Biopython. We identified 80 genes shared by all strains, including their annotations, in the database. Subsequently, genes with high copy numbers in the highly virulent strain HB03 were selected, resulting in 22 genes meeting the criteria. Finally, Vaxign2 and IEDB antibody epitope predictions were performed to analyze the properties of the proteins encoded by these genes, leading to the selection of nine candidate genes for vaccine development (**Table 4**). Furthermore, sequence alignment revealed that these nine genes were present in all 23 strains in the NCBI database (**Figure 1**).

**Table 4:** Copy number of candidate genes in 6 *M. synoviae* strains.

Description	HB01	HB02	HB03	HB04	WH01	WH02
class II fructose-1,6-bisphosphate aldolase	1	1	2	1	1	1
variable lipoprotein hemagglutinin VlhA	1	1	3	1	1	2
alanine–tRNA ligase	1	1	2	1	1	1
molecular chaperone DnaK	1	1	2	1	1	1
elongation factor Tu	1	1	2	1	1	1
transketolase	1	1	2	1	1	1
transposase	2	2	9	2	2	2
phosphopyruvate hydratase	1	1	3	1	1	1
elongation factor G	3	2	4	1	3	3

The VlhA protein consists of two variable cell surface proteins: variable lipoprotein (MSPB) and variable hemagglutinin protein (MSPA). Therefore, MSPA, MSPB, and eight other proteins were selected as candidate vaccine proteins in the subsequent experiments (**Table 5**).

**Table 5:** Information of ten candidate proteins.

Gene(ATCC 25204 strain WVU1853T)	Gene	Protein	Protein ID	Carrier	Protein size (kDa)
class II fructose-1,6-bisphosphate aldolase	cfba	Cfba	WP_020003203.1	pET-32a	51.64
Mycoplasma synoviae surface protein A	vlhA	MSPA	WP_082071168.1	pET-32a	43.12
Mycoplasma synoviae surface protein B	vlhA	MSPB	WP_082071168.1	pET-32a	53.46
alanine–tRNA ligase	ALAS	alanine–tRNA ligase	WP_011283519.1	pET-28a	101.18
Molecular chaperone	dnaK	DnaK	WP_020003200.1	pET-28a	70.07
Elongation factor Tu	tuf	EF-TU	WP_020003176.1	pET-32a	62.81
transketolase	TktA	transketolase	WP_011283343.1	pET-28a	72.54
transposase	DUF4277	transposase	ACL13536.1	pET-28a	65.01
Phosphopyruvate hydratase	eno	Ppht	WP_020003047.1	pET-32a	69.19
Elongation factor G	fusA	EF-G	WP_011283211.1	pET-28a	81.07

### 3.5 Immunoreactivity of candidate proteins with the MS-positive serum and MS- negative serum

**Figure 2:**
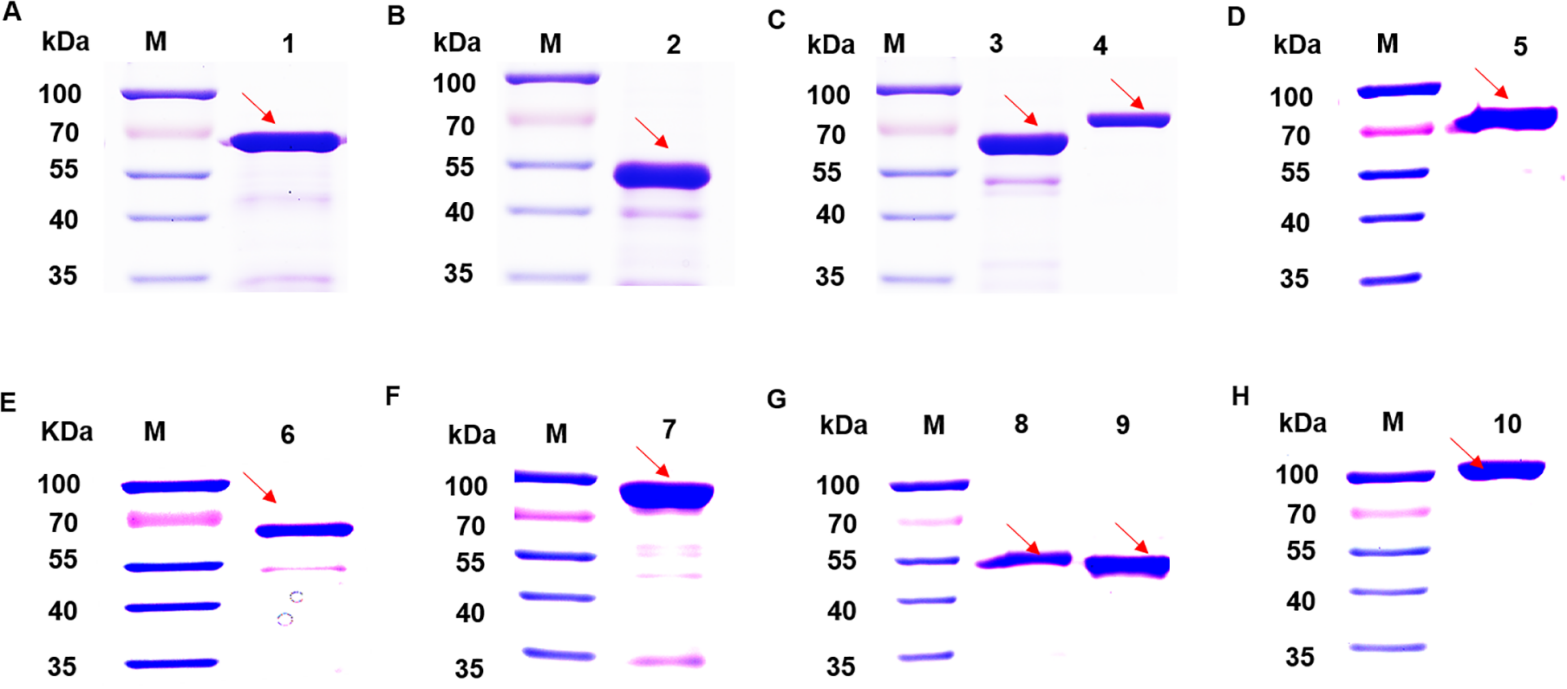
SDS-PAGE analysis of 10 candidate proteins. **(A)** Lane 1: transposase protein. **(B)** Lane 2: MSPA protein. **(C)** Lane 3: EF-TU protein. Lane 4: transketolase protein. **(D)** Lane 5: Dnak protein. **(E)** Lane 6:Ppht protein. **(F)** Lane 7: EF-G protein. **(G)** Lane 8: MSPB protein. Lane 9: Cfba protein. **(H)** Lane 10: alanine--tRNA ligase. The stripes indicated by the arrows are the intended bands for expression.

**Figure 3:**
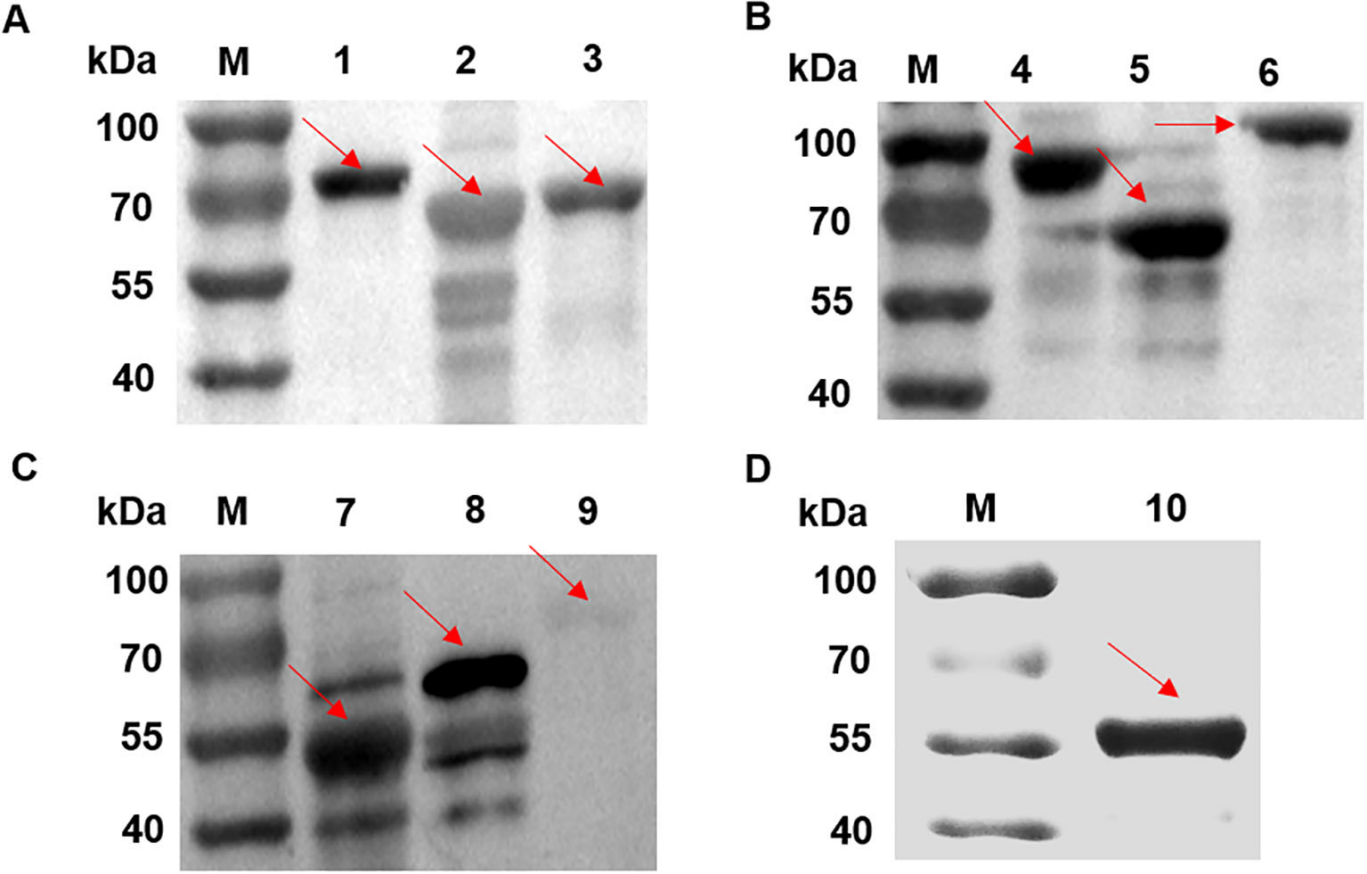
Western blot analysis of candidate proteins with MS-positive serum for immunogenicity identification. **(A)** Lane 1: transketolase protein. Lane 2: EF-TU protein. **(B)** Lane 3: transposase protein. Lane 4: EF-G protein. Lane 5: Ppht protein. **(C)** Lane 6: alanine–tRNA ligase. Lane 7: MSPA protein. Lane 8: Cfba protein. Lane 9: Dnak protein. **(D)** Lane 10: MSPB protein. The stripes indicated by the arrows showed that DnaK (Lane 9) did not react with the MS-positive serum, whereas the other nine proteins induced immune responses.

SDS-PAGE analysis of candidate proteins, all recombinant vectors expressed the target protein (**Figure 2**). Purified recombinant proteins were subjected to western blot analysis to assess their immunoreactivity with MS-positive serum and MS- negative serum. The results showed that DnaK did not react with the MS-positive serum, whereas the other nine proteins induced significant immune responses (**Figure 3**), while all recombinant proteins did not react with the MS- negative serum (**Supplementary Figure S1**), qualifying them as candidate vaccine proteins.

### 3.6 Evaluation of the immunoprotection ability of the candidate proteins

The immunoprotective efficacy of the nine proteins was assessed individually by establishing an infection model using the virulent strain HB03. The results revealed that the groups immunized a dose of 0.2mL with MSPB, Cfba, and EF-G proteins exhibited 30% protective efficacy. Ppht, EF-TU, and MSPA proteins showed 20% protective efficacy, while those immunized with alanine–tRNA ligase, transketolase protein and transposase protein showed 10% protective efficacy (**Table 6**). As none of the individual proteins achieved over 80% immunoprotection, further investigations will focus on combining MSPB, Cfba, EF-G, Ppht, EF-TU, and MSPA immunogenic proteins to identify combinations with high protective efficacies.

**Table 6:** Evaluation of the immune protection rate of individual candidate proteins.

No.	Gene name	Protein name	Group 2		Group 2	
			Immune dose	Protection rate (%)	Immune dose	Protection rate (%)
1	cfba	Cfba	0.1mL (20μg)	20%	0.2mL (40μg)	30%
2	vlhA	MSPB		20%		30%
3	eno	Ppht		20%		20%
4	tuf	EF-TU		20%		20%
5	fusA	EF-G		20%		30%
6	vlhA	MSPA		20%		20%
7	ALAS	alanine–tRNA ligase		10%		10%
8	TktA	transketolase		10%		10%
9	DUF4277	transposase		10%		10%
10	Adjuvant control	Adjuvant		0%		0%
11	Negative control	Modified Frey liquid culture medium		0%		0%

### 3.7 Evaluation of the immunogenicity of the combination vaccines

MSPB is an essential component of the key virulence gene *vlhA* in MS and its amino acid sequence is highly conserved. MSPB is associated with adhesion, invasion, and immune evasion in MS and is recognized as an immunogenic gene. Therefore, when grouping the six antigen proteins, MSPB was considered a mandatory component for each group. The combination groups comprised MSPB and Cfba, Ppht, EF-TU, EF-G, or MSPA. After immunization, protection tests against the virulent strain HB03 were conducted to screen for protein combinations with better synergistic effects.

#### 3.7.1 Immunogenicity of the two-component protein vaccines

As shown in **Table 7**, combining Cfba, Ppht, or EF-G with MSPB resulted in increased protective efficacy compared to the individual proteins, but it did not exceed 80% (**Table 7**). Therefore, further investigations of these combinations are necessary.

**Table 7:** Evaluation of the immune protection rate of dual-component candidate proteins.

Groups	Protein name	Symptoms				Any of the symptoms appearing	Protection rate (%)
		limping	Swollen foot pad	Swollen tarsal joint	Swollen toe joint		
Group1	MSPB,Cfba	40%	40%	20%	10%	60%	40%
Group2	MSPB,Ppht	50%	50%	20%	0%	60%	40%
Group3	MSPB,EF-TU	60%	60%	30%	60%	90%	10%
Group4	MSPB,EF-G	50%	50%	20%	0%	60%	40%
Group5	MSPB,MSPA	60%	60%	30%	60%	80%	20%
Adjuvant control	Adjuvant	70%	80%	20%	20%	100%	0%
Negative control	Modified Frey liquid culture medium	80%	80%	20%	20%	100%	0%

#### 3.7.2 Immunogenicity of multi-component protein vaccines

Continuing with the selection of Cfba, Ppht, EF-G, and MSPB for multi-component combinations, the results revealed that Immunization Group 1 (MSPB, Ppht, Cfba, and EF-G) exhibited a 100% immunization protection rate, Immunization Group 3 (MSPB, Cfba, and EF-G) demonstrated a 70% immunization protection rate, and Immunization Group 2 (MSPB, Ppht, and EF-G) and Immunization Group 4 (MSPB, Ppht, and Cfba) showed 60% immunization protection efficacy. Conversely, all chickens in the blank control group developed symptoms such as limping, foot swelling, toe joint swelling, and tarsal joint swelling. Using MSPB as the primary antigen, vaccines prepared with combinations of three proteins demonstrated an immunization protection rate of only 60–70%, which was insufficient to effectively shield against MS infection (**Table 8**). In contrast, the vaccine formulated with a combination of four proteins (MSPB, Ppht, Cfba, and EF-G) achieved a 100% immunization protection rate, qualifying it as a candidate combination for MS subunit vaccines.

**Table 8:** Evaluation of the immune protection rate of multi-component candidate proteins.

Groups	Protein name	limping	Swollen foot pad	Swollen tarsal joint	Swollen toe joint	Any of the symptoms appearing	Protection rate (%)
Group1	MSPB,Ppht,Cfba,EF-G	0%	0%	0%	0%	0%	100%
Group 2	MSPB,EF-G,Ppht	30%	30%	10%	10%	40%	60%
Group3	MSPB,EF-G,Cfba	30%	30%	0%	20%	30%	70%
Group 4	MSPB,Ppht,Cfba	40%	40%	0%	20%	40%	60%
Adjuvant control	Adjuvant	90%	90%	20%	20%	90%	10%
Negative control	Modified Frey liquid culture medium	100%	100%	100%	10%	90%	0%

### 3.8 The similarity of homologous protein sequences between MSPB, Cfba, Ppht, EF-G and those in the NCBI database

**Figure 4:**
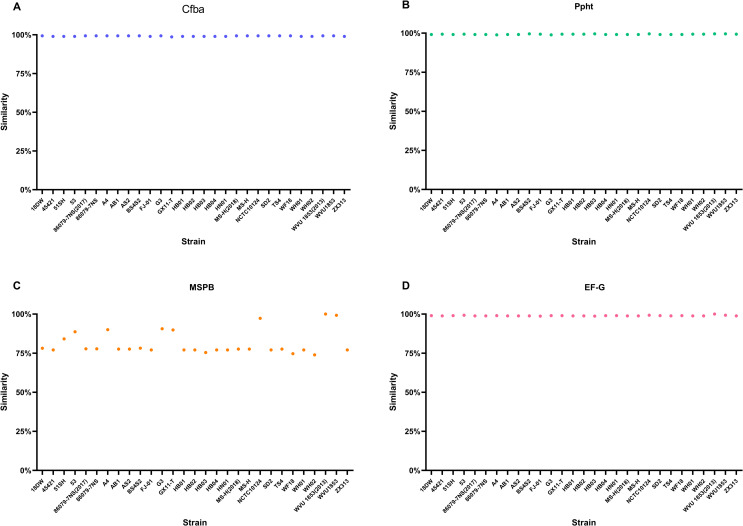
Similarity of homologous protein sequences between four vaccine candidate proteins and NCBI database. **(A)** Cfba. The similarity of Cfba ranges from 98.61% to 99.31%. **(B)** Ppht. The similarity of Ppht ranges from 98.89% to 99.56%. **(C)** MSPB. The similarity of MSPB ranges from 73.9% to 100%. **(D)** EF-G. The similarity of MSPB ranges from 98.71% to 100%.

Furthermore, MSPB, Ppht, Cfba, and EF-G involved in the vaccine developed in this study were present in all strains. The similarity of homologous protein sequences between Cfba, Ppht,EF-G and those in the NCBI database is approximately 98.6% to 100%, and the similarity of MSPB ranges from 73.9% to 100%, indicating a high degree of conservation and providing a basis for the broad-spectrum nature of the vaccine (**Figure 4**).

### 3.9 Dynamics of vaccine antibody decline and evaluation of the immunization protection duration

**Figure 5:**
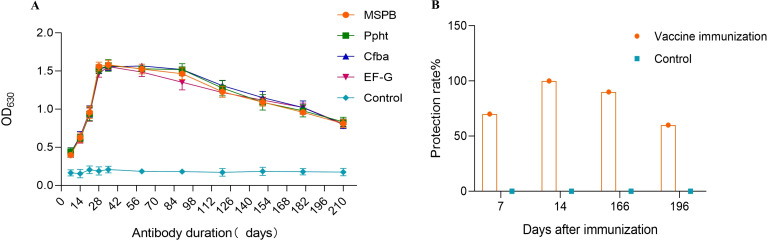
Duration of vaccine antibody (days) and Protection rate after 7, 14, 166, and 196 days of Second immunization. **(A)** Duration of vaccine antibody (days). Seven days after the initial immunization, some immunized chickens tended to have positive antibody levels. Antibody levels remained elevated from days 21 to 90, peaked at day 35, and then gradually decreased. **(B)** Protection rate after 7, 14, 166, and 196 days of Second immunization. 7 days after the second immunization, the protective efficacy against MS challenge was 70%. 14 days after the second immunization, the protective efficacy reached 100%. 166 days after the second immunization, the protective efficacy decreased to 90%. 196 days after the second immunization, the protective efficacy decreased further to 60%.

Monitoring the antibody decline pattern of the multi-component subunit vaccine revealed the following results. Seven days after the initial immunization, some immunized chickens tended to have positive antibody levels. Antibody levels remained elevated from days 21 to 90, peaked at day 35, and then gradually decreased (**Figure 5A**).

Testing the duration of immunization protection provided by the vaccine yielded the following findings. Twenty-one days after the initial immunization (7 days after the second immunization), the protective efficacy against MS challenge was 70%. 28 days after the initial immunization (14 days after the second immunization), the protective efficacy reached 100%. 180 days after the initial immunization (166 days after the second immunization), the protective efficacy decreased to 90%. 210 days after the initial immunization (196 days after the second immunization), the protective efficacy decreased further to 60% (**Figure 5B**).

Therefore, the immune production period of the subunit vaccine against *Mycoplasma synoviae* in chickens is determined to be 28 days after the first immunization, and the immune duration is 180 days.

### 3.10 Analysis of pathological changes in tissue organs of vaccinated chickens

**Figure 6:**
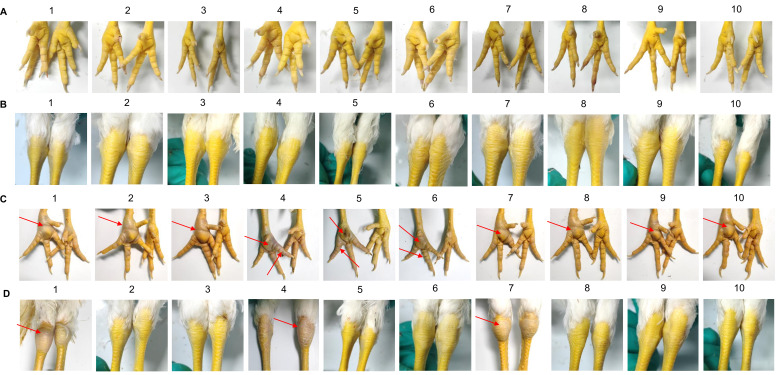
The symptoms of palmar, toe joints and tarsal of the post immunization and non immunization challenge groups. **(A)** The symptoms of palmar, toe joints of the post immunization challenge groups. 10 SPF chickens in the post immunization challenge groups, were found to have normal foots and toe joints. **(B)** The symptoms of tarsal of the post immunization challenge groups. 10 SPF chickens in the post immunization challenge groups, were found to have normal tarsals. **(C)** The symptoms of palmar, toe joints of the non immunization challenge groups. 10 SPF chickens in the non immunization challenge groups, were found to have swollen foot. **(D)** The symptoms of tarsal of the post immunization challenge groups. 10 SPF chickens in the non immunization challenge groups, were found to have swollen tarsal.

**Figure 7:**
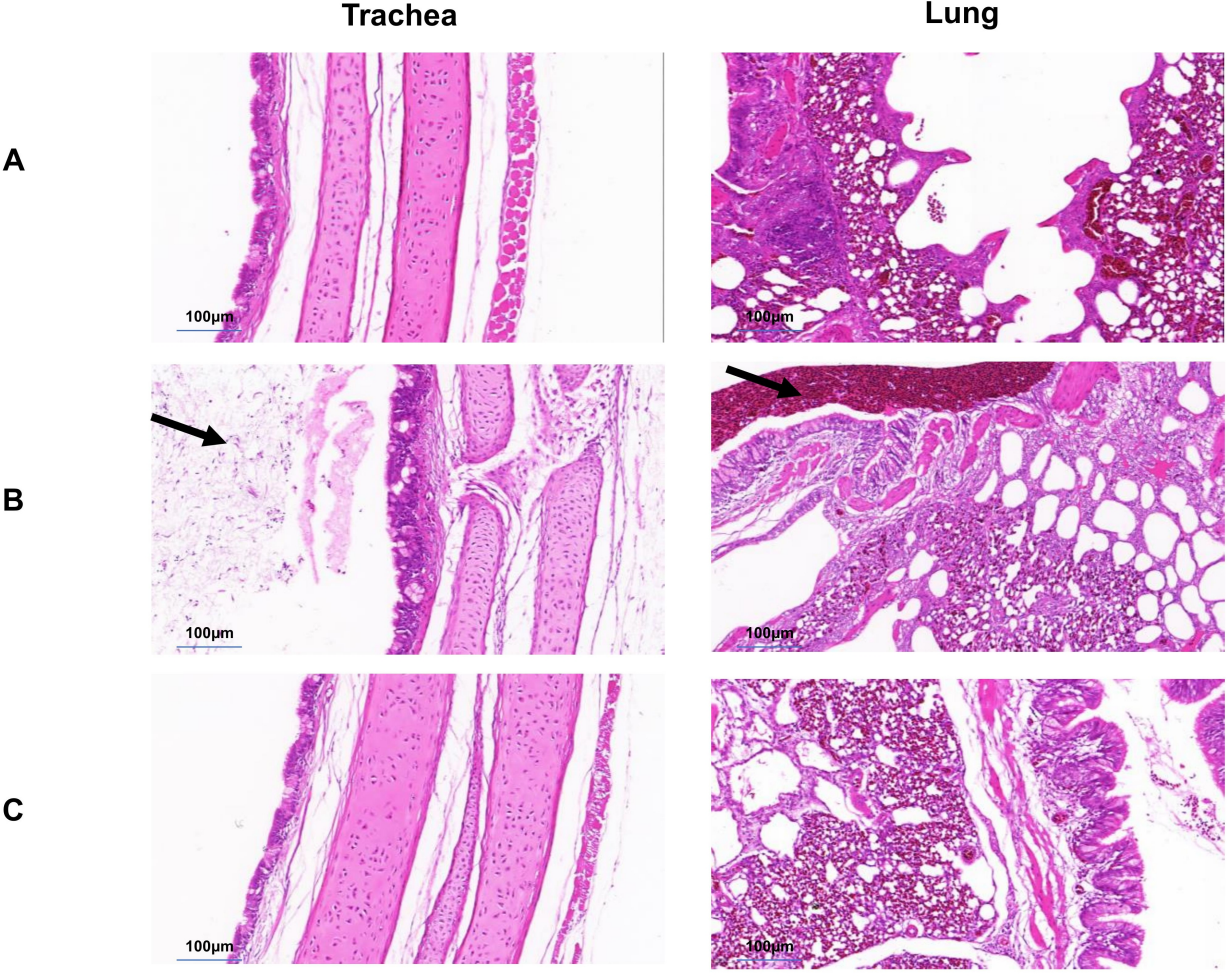
Histological examination (H&E staining) of trachea and lung. **(A)** Normal group. No abnormalities were found in the trachea and lungs. **(B)** Unimmunized group. Mucus, serous fluid, and inflammatory cell exudation can be seen on the surface and lumen of the tracheal mucosa; A large number of red blood cells and exfoliated cells can be seen in the lumen of the secondary bronchial tube of the lung **(C)** Immune group. No abnormalities were found in the trachea and lungs.

After the 0.2mL dose of the immune group was administered, 10 SPF chickens in the post immunization challenge groups, were found to have normal foot, tarsal, and toe joints Compared with the non immunization challenge groups (**Figure 6**). Histopathological examination of the trachea and lung from the the post immunization challenge groups revealed no significant abnormalities, same as the normal group (**Figure 7**).

## 4 Discussion

Although chicken MS has only one serotype, significant differences in pathogenicity and immunity exist among different clinical isolates, rendering existing live attenuated and inactivated vaccines ineffective in providing broad cross-protection. In this study, we compared the whole-genome sequences of strongly and weakly pathogenic strains and identified nine genes that were common to all strains and positively correlated with virulence as candidate antigen-encoding genes for a vaccine. These genes included *vlhA*, *eno*, *cfba*, and *fusA*. Subsequently, we successfully developed a highly efficient multi-component subunit vaccine.

Studies have indicated that the *vlhA*, *cfba*, and *eno* genes are associated with MS virulence. Among these, *vlhA* encodes the variable lipoprotein hemagglutinin (VlhA) protein, which plays a significant role in aiding MS in adhering to host cells and evading the immune system (Jeffery et al., 2006; Slavec et al., 2011). Following ribosomal translation, this protein is cleaved into the C-terminal (MSPA) and N-terminal (MSPB) domains. MSPB interacts with the host cell membrane via its C-terminal end, facilitating the adherence of MS to sialylated cell receptors on the host. This is currently considered the primary mechanism underlying the pathogenicity of MS (Lavric et al., 2007).

Ppht, which is encoded by the *eno* gene, is a phosphohexose isomerase that plays a crucial role in the metabolism of MS and may influence the infection and immune response to MS (Bao et al., 2014). Studies have indicated that the α-enolase proteins of avian mycoplasmas are involved in the adherence to DF-1 cells (Chen et al., 2011). Additionally, research on other pathogens has suggested that enolases participate in the adhesion of pathogens to host cells and serve as adhesion factors (Marcos et al., 2012; Nogueira et al., 2012; Song et al., 2012).

The *cfba* gene encodes fructose 1,6-bisphosphate aldolase, which is widely present in animals, plants, and microorganisms. This protein can bind to the cytoskeleton, participate in microtubule polymerization, and is involved in pathogen invasion (Gao et al., 2018; Yu et al., 2018; Huang et al., 2019). Therefore, the genes identified by the screening method used in this study were likely positively correlated with the virulence of MS.

This study developed a highly efficient subunit vaccine, composed of MSPB, Ppht, Cfba, and EF-G. These four proteins were present in all reported viral sequences and have a high degree of similarity, indicating a high degree of conservation and providing a basis for the broad-spectrum nature of the vaccine.However, the subunit vaccine included four antigenic proteins, which may increase the complexity of vaccine production and hinder its widespread application. Subsequent research should focus on further analyzing the core antigenic epitopes to reduce the variety of antigenic proteins through tandem expression, thereby optimizing the preparation process and cost of the vaccine.

In summary, this study utilized whole-genome sequencing analysis to screen for proteins common to clinically isolated MS strains and positively correlated with virulence as candidate antigens for vaccine production. A multi-component subunit vaccine against MS was successfully developed, which was capable of generating over 90% protection against virulent strains. Ultimately, this vaccine may serve as a candidate for preventing MS-induced disease, providing a potentially effective means for MS control.

## Data Availability

All data generated or analyzed during this study are included in the article. And the genome accessions are included below and are present in the “genome info” file of this submission at https://submit.ncbi.nlm.nih.gov/subs/genome/.

## Supplementary material

Supplementary Figure 1 Western blot analysis of candidate proteins with MS-negative serum for immunogenicity identification. **(A)** Lane 1: MSPA protein. Lane 2: MSPB protein. Lane 3: alanine–tRNA ligase protein. Lane4: transketolase protein. **(B)** Lane 5: Cfba protein. Lane 6: Dnak protein. Lane 7: EF-TU protein. **(C)** Lane 8: EF-G protein. Lane 9: transposase protein. Lane 10: Ppht protein. All proteins can not react with MS-negative serum.
