# Supplementary figures and images for: Target antigen screening and development of a multi-component subunit vaccine against Mycoplasma synoviae in chickens
Source: Front Cell Infect Microbiol. 2024 Oct 23;14:1458865. doi: 10.3389/fcimb.2024.1458865 (PMC11537996; doi:10.3389/fcimb.2024.1458865)

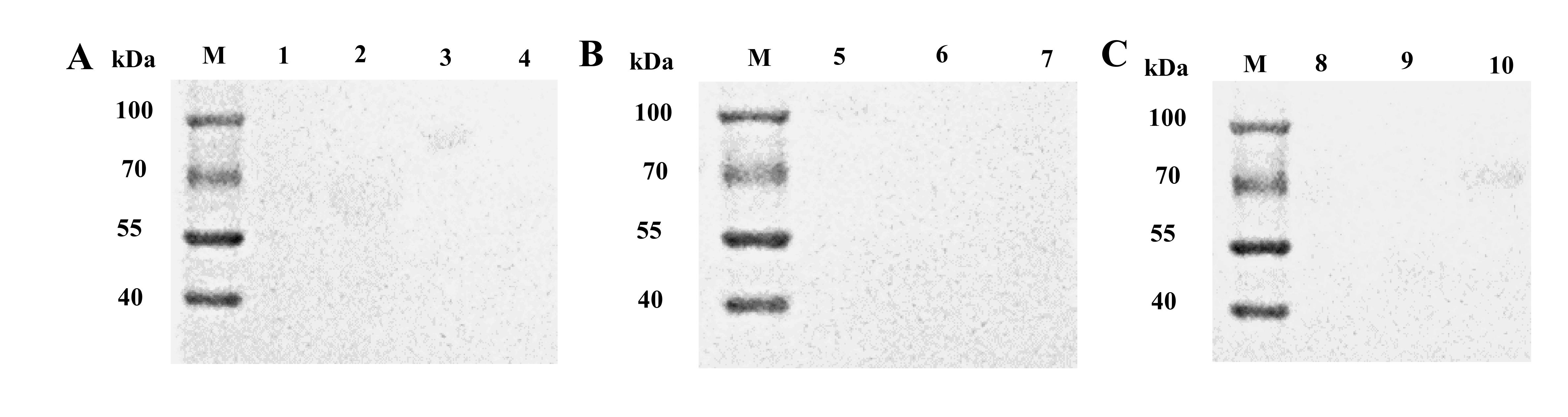

Supplement: Supplementary Figure 1 — Western blot analysis of candidate proteins with MS-negative serum for immunogenicity identification. (A) Lane 1: MSPA protein. Lane 2: MSPB protein. Lane 3: alanine–tRNA ligase protein. Lane4: transketolase protein. (B) Lane 5: Cfba protein. Lane 6: Dnak protein. Lane 7: EF-TU protein. (C) Lane 8: EF-G protein. Lane 9: transposase protein. Lane 10: Ppht protein. All proteins can not react with MS-negative serum. [file Image1.tif]
